# Statin use in cirrhotic patients with infectious diseases: A population-based study

**DOI:** 10.1371/journal.pone.0215839

**Published:** 2019-04-24

**Authors:** Tsung-Hsing Hung, Chih-Chun Tsai, Hsing-Feng Lee

**Affiliations:** 1 Department of Medicine, Dalin Tzu Chi Hospital, Buddhist Tzu Chi Medical Foundation, Chiayi, Taiwan; 2 School of Medicine, Tzu Chi University, Hualien, Taiwan; 3 Department of Mathematics, Tamkang University, Tamsui, Taiwan; University of Manitoba, CANADA

## Abstract

**Background:**

Recent studies have shown benefits of statins in patients with liver cirrhosis. However, it is still unknown if statins have a beneficial effect on the mortality of cirrhotic patients with bacterial infections.

**Methods:**

The Taiwan National Health Insurance Database was searched, and 816 cirrhotic patients receiving statins with bacterial infections hospitalized between January 1, 2010 and December 31, 2013 were included in the study. A one-to-four propensity score matching was performed to select a comparison group based on age, sex, and comorbid disorders.

**Results:**

The overall 30-day mortalities in statin and non-statin group were 5.3% and 9.8%, respectively (P = 0.001). After Cox regression modeling adjusting for age, sex, and comorbid disorders, the hazard ratio (HR) of statin use on 30-day mortality was 0.52 (95% confidence interval [CI]: 0.38–0.72, P<0.001). In subgroup analysis, the 30-day mortality effect of statin use was more pronounced in patients with pneumonia (HR = 0.34; 95% CI: 0.19–0.59; P<0.001) and bacteremia (HR = 0.55; 95% CI: 0.35–0.85; P = 0.008). Atovastatin (HR = 0.59; 95% CI: 0.37–0.93) and rosuvastatin (HR = 0.59; 95% CI: 0.36–0.98) were associated with a decreased 30-day mortality risk compared to patients not taking statins.

**Conclusions:**

Statin use decreases the 30-day mortality of cirrhotic patients with bacteremia and pneumonia.

## Introduction

Bacterial infections are the major cause of hospitalization for patients with liver cirrhosis [1]. The mortality of patients with cirrhosis and bacterial infections is increased about four-fold during hospitalization [2]. In addition, bacterial infections can trigger or aggravate cirrhosis-related complications such as hepatic encephalopathy, ascites, or variceal bleeding [1–6], all of which may further increase the mortality of cirrhotic patients.

Statins are usually for the treatment of dyslipidemia or various cardiovascular diseases. However, other possible benefits of statins have been evaluated. Statin use could cause the liver damage occasionally. The potentially liver toxicity of statins have led to increasing concern by physicians. Although recent studies have shown beneficial effect of statins in cirrhotic patients [7–13], the effect of statins on bacterial infections in different population is unclear [14–16]. As such, examining the effect of statins on the mortality of cirrhotic patients with bacterial infections is an important area of research. Thus, the purpose of this study was to use the Taiwan National Health Insurance Database to examine the outcomes of hospitalized cirrhotic patients receiving statins with bacterial infections. Propensity score matching was performed to select a comparison group based on their age, sex, and comorbid disorders, socioeconomic status, or etiology of liver cirrhosis. In the subgroup analysis, we also calculate the hazard ratios of risk factors of statins for 30-day mortalities among cirrhotic patients with different bacterial infections, such as pneumonia, spontaneous bacterial peritonitis, urinary tract infection, or bacteremia. In this study, different statins were also evaluated for the 30-day mortalities in cirrhotic patients, compared to non-statin users.

## Materials and methods

### Database and ethical statement

The National Health Insurance Administration (NHIA) in Taiwan instituted a National Health Insurance program that covers more than 99% of the Taiwan population. In this program, all the enrolled medical institutions must provide medical records to the NHIA for medical payments. The medical records were established as a database, the Taiwan National Health Insurance Research Database (NHIRD). This database includes International Classification of Diseases, 9th Revision, Clinical Modification (ICD-9-CM) codes, medical procedures, hospitalization days, drugs, and other pertinent information of patients in Taiwan who have been hospitalized.

We used a dataset from the NHIRD to perform this study (application and agreement number 104359). This study was approved by the Institutional Review Board of the Buddhist Dalin Tzu Chi Hospital (IRB B10403026). Because all of the data in the NHIRD is de-identified, the review board waived the requirement of informed patient consent.

### Study sample

The database was searched for patients discharged between January 1, 2010 and December 31, 2013 with a primary or accessory diagnosis of cirrhosis (ICD-9-CM code 571.5 or 571.2). These ICD-9 codes have been used in past studies to identify patients with cirrhosis in Taiwan [17, 18]. This group of patients was then searched for those with bacterial infections.

The bacterial infections included were bacteremia (ICD-9-CM code 038, 020.2, 790.7, or 112.81), cellulitis (ICD-9-CM code 681 or 682), pneumonia (ICD-9-CM code 481–487, without 484), biliary tract infection (BTI) (ICD-9-CM code 576.1, 575.0, 574.00, 574.01, 574.30, 574.31, 574.60, 574.61, 574.80, 574.81), necrotizing fasciitis (NF) (ICD-9-CM code 728.86), empyema (ICD-9-CM code 510), brain abscess (ICD-9-CM code 324), urinary tract infection (UTI) (ICD-9-CM code 590.1, 595.0, 595.9 or 599.0), septic arthritis (SA) (ICD-9-CM code 711), perianal abscess (ICD-9-CM code 566), liver abscess (ICD-9-CM code 572.0), bacterial meningitis (ICD-9-CM code 320), and spontaneous bacterial peritonitis (SBP) (ICD-9-CM codes 567.2, 567.8, or 567.9. Patients with other diagnostic coding for secondary peritonitis, such as appendicitis, ischemic bowel disease, peritoneal dialysis catheter-related peritonitis, hollow organ or biliary tract perforation, or those having an additional procedure code for abdominal surgery were excluded from the study [18]. If a patient had multiple hospitalizations for an infection during the study period, only the first episode was included in the analysis. Of these patients, those taking statins including atovastatin, rosuvastatin, fluvastatin, simvastatin, pravastatin, lovastatin, and pitavastatin were considered the statin group. One-to-four propensity score matching was used to select a non-statin group according to age, sex, socioeconomic stat (SES), and comorbid disorders including the etiology of liver cirrhosis (alcoholism (ICD-9-CM codes 291, 303, 305.00–305.03, 571.0–571.3), chronic hepatitis B, or chronic hepatitis C), hepatocellular carcinoma (ICD-9-CM code 155.0), diabetes mellitus (DM) (ICD-9-CM code 250, or receiving insulin or oral hypoglycemic agents),renal function impairment (ICD-9-CM code 584, 585, 586, 572.4, or other procedure codes relate to renal failure), liver reserve, and steroid use. The individuals were classified into three groups: low SES, medium SES, and high SES. In this study, low SES was defined as monthly income lower than New Taiwan Dollar (NTD $ 20000) (about US$ 556). Medium SES was defined as monthly income between NTD $20001–40000 (about US$ 556–1111). High SES was defined as monthly income more than NTD $ 40001 (about US$ 1111). In this study, the liver reserve was defined the presence of number of cirrhotic-related complications (variceal bleeding, hepatic encephalopathy, or ascites) during hospitalization.

### Statistical analyses

The SPSS statistical package version 22.0 for Windows was used to analyze the data. The chi square test was used to compare categorical variables, and Student’s t test was used to compare continuous variables. The proportional hazards Cox regression model was used to evaluate the comorbid factors, with reporting of hazard ratios (HRs) and 95% confidence intervals (CIs). The significance level was set at 0.05.

## Results

After review of the database and application of the inclusion and exclusion criteria 816 patients with cirrhosis with bacterial infections receiving statins (statin group) were included in the study. After 1:4 propensity score matching, 3,264 cirrhotic patients with infections who were no receiving statins were included as the non-statin group. Table 1 shows the demographic characteristics of the statin and non-statin groups. The overall 30-day mortalities for the statin group and non-statin group were 5.3% and 9.8%, respectively (P<0.001). After Cox regression modeling adjusting for age, sex, and other comorbid disorders, the HR for 30-day mortality of the statin group was 0.52 (95% CI, 0.38–072, P<0.001) as compared to the non-statin group. Other statistically significant prognostic factors are summarized in Table 2. Age, etiology of liver cirrhosis, liver reserve, RFI, and steroid and statin usage were associated with significant differences in 30-day overall mortality.

**Table 1:** Demographic characteristics of the statin and non-statin groups.

	Statin group (n = 816)	Non-statin group (n = 3264)	P value
Male	467 (57.2)	1947 (59.7)	0.208
Age, y	66.28 ± 14.03	65.91 ± 14.78	0.526
HCC	53 (6.5)	195 (6.0)	0.578
Complication conditions			
No complication	737 (90.3)	2957 (90.6)	0.810
1 complication	71 (8.7)	274 (8.4)	0.778
2 or 3 complications	8 (1.0)	33 (1.0)	0.937
RFI	116 (14.2)	455 (13.9)	0.839
DM	551 (67.5)	2177 (66.7)	0.653
Etiology			
Alcoholism	69 (8.5)	284 (8.7)	0.824
HBV	113 (13.8)	436 (13.4)	0.714
HCV	99 (12.1)	369 (11.3)	0.507
Steroid	220 (27.0)	876 (26.8)	0.944
Socioeconomic status			
Low	289 (35.4)	1217 (37.3)	0.322
Medium	421 (51.6)	1652 (50.6)	0.616
High	106 (13.0)	395 (12.1)	0.489

Age presented as mean ± standard deviation; other data as number (percentage).

Abbreviations: HCC, hepatocellular carcinoma; HE, hepatic encephalopathy; EVB, esophageal variceal bleeding; RFI, renal function impairment; DM, diabetes mellitus.

**Table 2:** Adjusted hazard ratios of risk factor for 30-day mortality of cirrhotic patients with bacterial infections.

Variable	Hazard ratio	95% Confidence Interval	P value
Male	1.18	0.94–1.48	0.155
Age, y	1.01	1.00–1.02	0.018
HCC	2.83	2.09–3.83	<0.001
Complication conditions			
No complication			<0.001
1 complication	2.68	2.05–3.50	<0.001
2 or 3 complications	3.80	2.07–6.98	<0.001
RFI	2.40	1.91–3.02	<0.001
DM	1.28	1.01–1.63	0.041
Etiology of cirrhosis			
Alcoholism	1.39	0.98–1.98	0.067
HBV	0.32	0.19–0.53	<0.001
HCV	0.16	0.08–0.35	<0.001
Steroid	2.55	2.06–3.15	<0.001
Socioeconomic status			
Low			0.496
Medium	1.07	0.86–1.34	0.539
High	0.87	0.61–1.26	0.470
Statin	0.52	0.38–0.72	<0.001

Abbreviations: HCC, hepatocellular carcinoma; HE, hepatic encephalopathy; EVB, esophageal variceal bleeding; RFI, renal function impairment; DM, diabetes mellitus.

**Fig 1:**
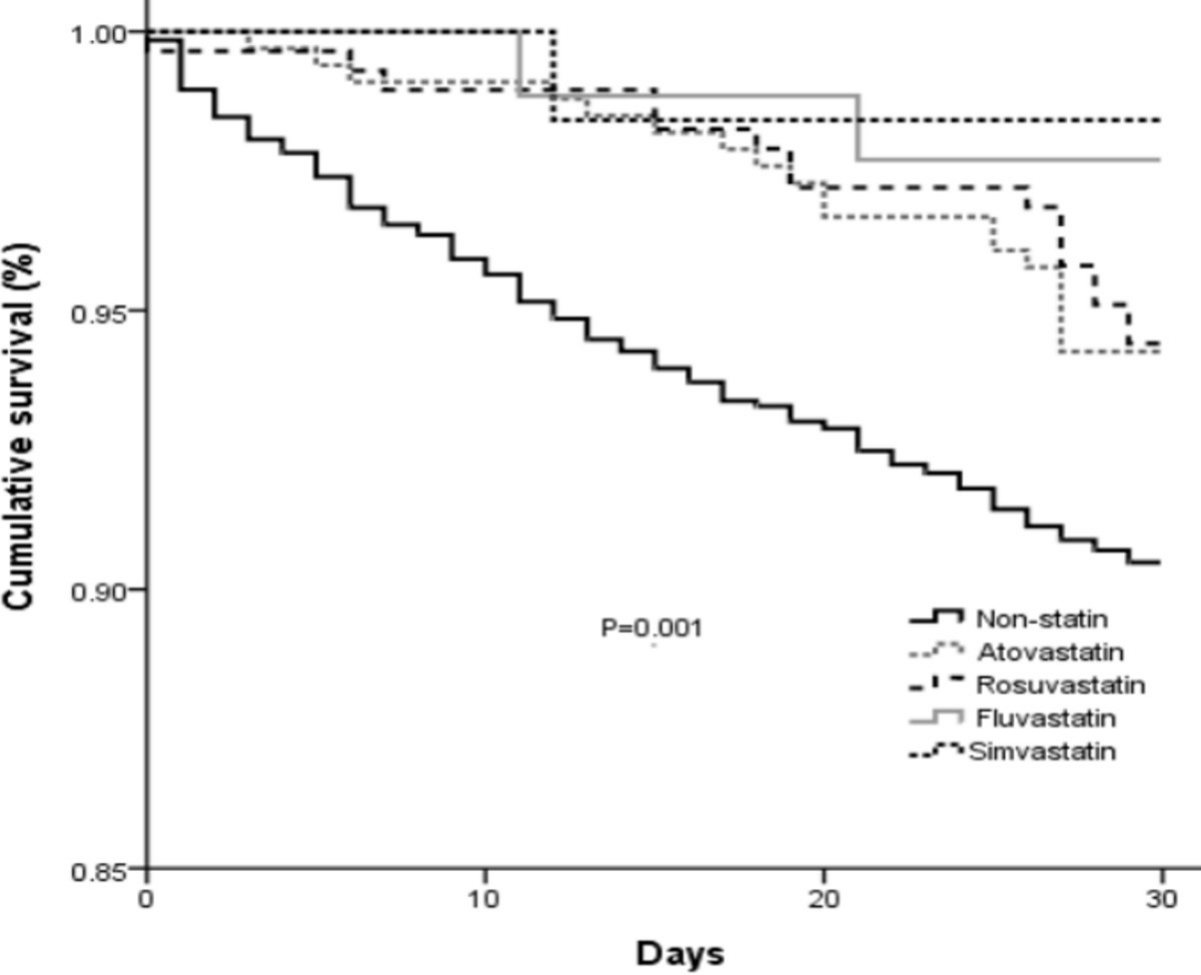
Kaplan–Meier survival analysis of 30-day mortally of cirrhotic patients with bacterial infections using different kinds of statins, compared to non-statin users.

To evaluate the effect of each kind of statin on the mortality of cirrhotic patients with bacterial infections, each of the individual statins were compared to the non-statin group. Because there were only a few patients receiving pravastatin, lovastatin, or pitavastatin, these were not included in the regression analysis. Results of the analysis are shown in Table 3. Oral atovastatin and rosuvastatin were associated with a decreased 30-day mortality risk. The 30-day mortality risks of patients taking fluvastatin and simvastatin were not different compared to the non-statin group. The cumulative survival plots for the individual statins are shown in Fig 1.

**Table 3:** Adjusted hazard ratios of different statins for 30-day mortalities of cirrhotic patients with bacterial infections, compared to non-statin users.

	Case/control	HR (95% CI)	P value
Statin	816/3264	0.52 (0.38–0.72)	<0.001
Atovastatin	332/3264	0.59 (0.39–0.93)	0.024
Rosuvastatin	286/3264	0.59 (0.36–0.98)	0.040
Fluvastatin	87/3264	0.30 (0.07–1.20)	0.088
Simvastatin	63/3264	0.19 (0.03–1.38)	0.101

Abbreviations: HR, hazard ratio; CI, confidence interval.

**Fig 2:**
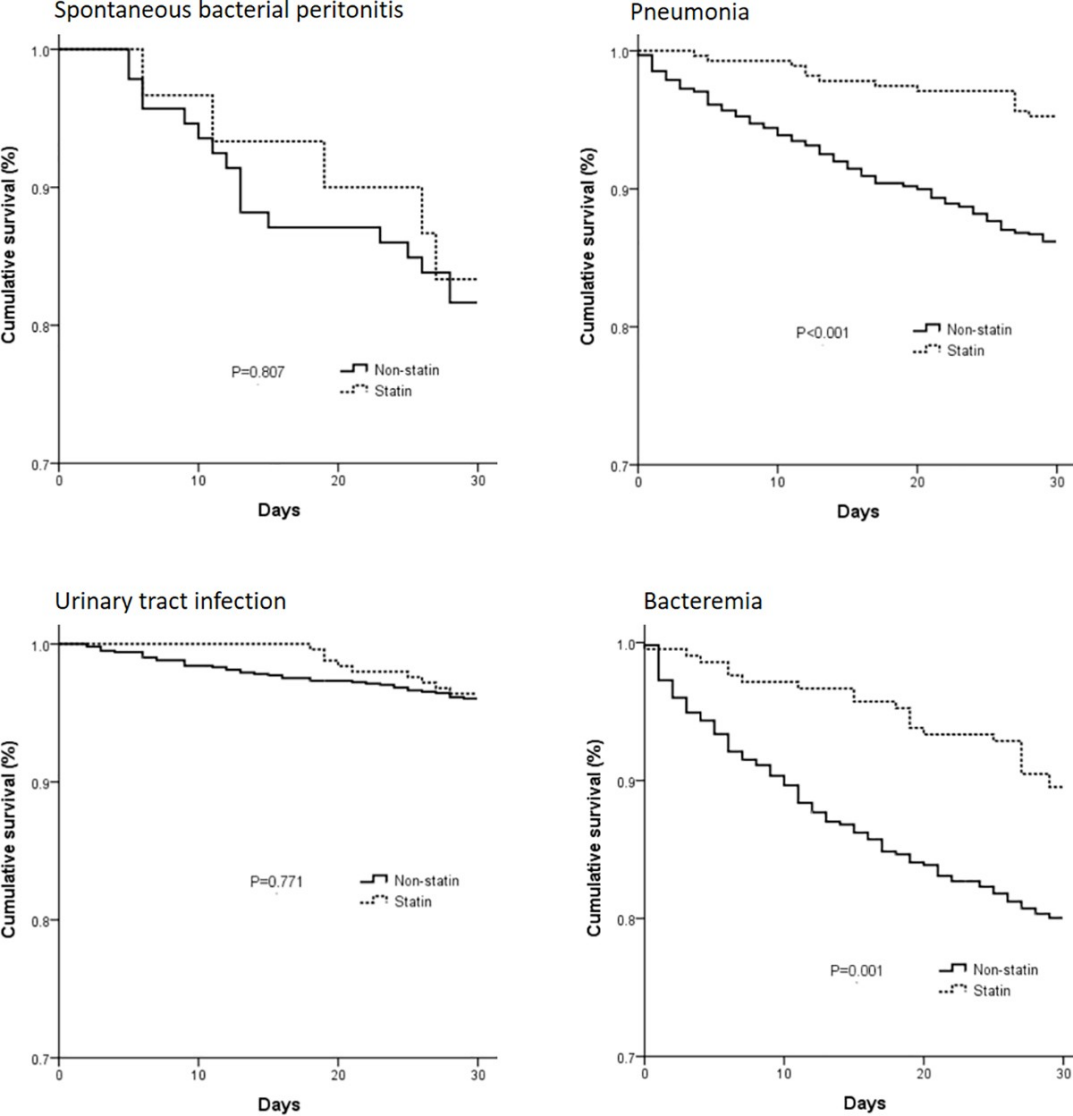
Kaplan–Meier survival analysis of 30-day mortality of cirrhotic patients with different bacterial infections.

We next stratified all patients based on four major infectious diseases in cirrhotic patients: SBP, pneumonia, UTI, and bacteremia. There were 275 patients with pneumonia in the statin group, and 948 in the non-statin group. Cox regression modeling adjusting for age, sex, SES, and other comorbid disorders found that statins had a beneficial effect on 30-day mortality in patients with pneumonia (HR = 0.34; 95% CI: 0.19–0.59, P≤0.001). There were 210 patients with bacteremia in the statin group, and 1,024 in the non-statin group. Cox regression modeling showed that statins had a beneficial effect on 30-day mortality of patients with bacteremia (HR = 0.55; 95% CI: 0.35–0.85; P = 0.008). No benefit of statins on 30-day mortality in patients with SBP and UTI group was found. Regression analysis results are summarized in Table 4, and cumulative survival plots for each type of infection are shown in Fig 2. The age of 30-day mortalities for the statin group and non-statin group were calculated and the results were provided in Table 5. The stains significantly decrease the 30-day mortality in cirrhotic patients more than 50 years old.

**Table 4:** Adjusted hazard ratios of risk factor of statins for 30-day mortalities of cirrhotic patients with different bacterial infections.

	Case/control	HR (95% CI)	P value
SBP (n = 123)	30/93	0.84 (0.29–2.49)	0.757
Pneumonia (n = 1223)	275/948	0.34 (0.19–0.59)	<0.001
UTI (n = 1262)	250/1012	0.89 (0.43–1.86)	0.762
Bacteremia (n = 1234)	210/1024	0.55 (0.35–0.85)	0.008

Abbreviations: SBP, spontaneous bacterial peritonitis; UTI, urinary tract infection; HR, hazard ratio; CI, confidence interval

**Table 5:** The age of 30-day mortalities for the statin group and non-statin group.

Age, y (case numbers)	Statin /Non-statin (case numbers)	30-day mortality (%)		P value
		Statin group	Non-statin group	
< 40 (n = 156)	32/124	3.1	7.3	0.393
40–49 (n = 462)	79/383	5.1	7.3	0.122
50–59 (n = 795)	148/647	2.7	8.4	0.018
60–69 (n = 786)	176/610	4	9.2	0.024
>70 (n = 1881)	381/1500	6.8	10	0.049

## Discussion

Statins are usually for the treatment of dyslipidemia, and are now also used for the prevention or treatment of various cardiovascular diseases. However, other possible benefits of statins have been the topic of current research. Although there is the potential of hepatotoxicity in statin users with chronic liver disease, the toxicity is usually mild and well tolerated. The safety of satins in patients with chronic liver disease has been shown in a prior study [19]. A number of current studies have shown beneficial effects of statins in cirrhotic patients [7–13]. However, the effect of statins in patients with cirrhosis and bacterial infections is unclear [14–16]. Statins have been shown to have anti-inflammatory effects, and are associated with reducing or preventing the risk of liver fibrosis progression in patients with chronic liver disease [20]. Statins have also been shown to decrease the hepatic venous pressure gradient and improve liver perfusion in patients with cirrhosis [21]. A prior study found that patients with cirrhosis taking statins experienced hospitalizations with infections at a rate 0.67 less than that of non-users. [22] However, the effect of statins on mortality of cirrhotic patients with bacterial infections is not clear.

In our study with propensity score matching, we demonstrate that statins were associated with lower risk of mortality in patients with cirrhosis and bacterial infections as compared to patients who were not taking statins. This beneficial effect of statins has been shown in prior studies [11,22]. A prior meta-analysis showed that statin use was associated with a 42% reduction in the risk of developing cirrhosis in Eastern and Western countries [12]. Another meta-analysis showed that pooled HR for the progression of hepatic fibrosis for patients taking statins was 0.49 as compared to those not taking statins [23].

An important point about the current population-based study is that statins were being used to treat dyslipidemia and various cardiovascular diseases in the vast majority of patient. However, as pointed out in a prior study statin use cannot be recommended for use in all cirrhotic patients [11]. On the other hand, clinicians should understand that cirrhosis is not a contraindication to statins in patients with dyslipidemia or other cardiovascular diseases. In this study, the socioeconomic status is not the factor for 30-day mortality. In Taiwan, liver cirrhosis is considered as a catastrophic illness in our health insurance program. Almost the medical payment can be covered by the health insurance program. This is the reason why socioeconomic status is not the factor for short-term mortality in our study.

Our analysis of individual statins showed that atovastatin and rosuvastatin were associated with a significantly decreases risk of 30-day mortality risk in cirrhotic patients with bacterial infections. However, these results were not found with other statins and this is presumably due to small numbers of patients taking other statins. We also evaluated the effect of statins in patients with different types of bacterial infections, and found that statins decreased the 30-day mortality of patients with pneumonia and bacteremia. Comparing to other bacterial infectious diseases in cirrhotic patients, UTIs are considered a less severe infection. In the current study, the 30-day mortality of patients with UTIs in the statin and non-statin group was only 3.6% and 4.0%, respectively. The finding of no difference between the statin and non-statin group may be due to the overall low mortality rate associated with UTIs. There was also no difference in mortality in patients with SBP, and this may be because there were only 30 patients with cirrhosis and SBP.

While this study demonstrated an important role of statins in cirrhotic patients with bacterial infection, there are several limitations that need to be considered regarding this population-based study. First, data of the Mayo Clinic model for end-stage liver disease (MELD) score and Child-Pugh score were not available. This is an essential disadvantage because no laboratory data such as bilirubin level, albumin level, or prothrombin time were available in the dataset. In this study, the stage of liver cirrhosis was defined the presence of number of cirrhosis-related complications (variceal bleeding, hepatic encephalopathy, or ascites) during hospitalization. The major cirrhosis-related complications were considered in the regression analyses for mortality risk. These clinical factors of liver cirrhosis have been considered important factors for staging liver cirrhosis [24,25]. Third, the reason for receiving statin could not be clarified in this study. People who use statins may be wealthier, so the socioeconomic status was considered in this study, even by propensity score matching or regression analysis. Lastly, the duration of exposure to statins was not known. We could not understand the duration of statin usage before or after hospitalizations from the dataset we applied.

In conclusion, this nationwide population-based study showed that statins can decrease the 30-day mortality of cirrhotic patients with bacteremia or pneumonia. Although we cannot recommend the routine use of statins in cirrhotic patients, statins should not be considered a contraindication in cirrhotic patients, even those with pneumonia or bacteremia.

## References

[1] Jalan R, Fernandez J, Wiest R. Bacterial infections in cirrhosis: a position statement based on the EASL Special Conference 2013. J Hepatol 2014;60:1310–1324. 10.1016/j.jhep.2014.01.024 24530646

[2] Arvaniti V, D'Amico G, Fede G, Manousou P, Tsochatzis E, Pleguezuelo, et al Infections in patients with cirrhosis increase mortality four-fold and should be used in determining prognosis. Gastroenterology 2010;139:1246–1256. 10.1053/j.gastro.2010.06.019 20558165

[3] Bonnel AR, Bunchorntavakul C, Reddy KR. Immune dysfunction and infections in patients with cirrhosis. Clin Gastroenterol Hepatol 2011;9:727–738. 10.1016/j.cgh.2011.02.031 21397731

[4] Kang SH, Lee YB, Lee JH, Nam JY, Chang Y, Cho H, et al Rifaximin treatment is associated with reduced risk of cirrhotic complications and prolonged overall survival in patients experiencing hepatic encephalopathy. Aliment Pharmacol Ther 2017;46:845–855. 10.1111/apt.14275 28836723

[5] Fernández J, Tandon P, Mensa J, Garcia-Tsao G. Antibiotic prophylaxis in cirrhosis: Good and bad. Hepatology 2016; 63:2019–2031. 10.1002/hep.28330 26528864

[6] Chang CJ, Hou MC, Liao WC, Chen PH, Lin HC, Lee FY, et al Management of acute gastric varices bleeding. J Chin Med Assoc 2013;76:539–546. 10.1016/j.jcma.2013.06.011 23880574

[7] Chang FM, Wang YP, Lang HC, Tsai CF, Hou MC, Lee FY, et al Statins decrease the risk of decompensation in hepatitis B virus- and hepatitis C virus-related cirrhosis: A population-based study. Hepatology 2017;66:896–907. 10.1002/hep.29172 28318053

[8] Kim RG, Loomba R, Prokop LJ, Singh S. Statin use and risk of cirrhosis and related complications in patients with chronic liver diseases: a systematic review and meta-analysis. Clin Gastroenterol Hepatol 2017;15:1521–1530. 10.1016/j.cgh.2017.04.039 28479502 PMC5605397

[9] Huang YW, Lee CL, Yang SS, Fu SC, Chen YY, Wang TC, et al Statins reduce the risk of cirrhosis and its decompensation in chronic hepatitis B patients: a nationwide cohort study. Am J Gastroenterol 2016;111:976–85. 10.1038/ajg.2016.179 27166128

[10] Simon TG, Bonilla H, Yan P, Chung RT, Butt AA, et al Atorvastatin and fluvastatin are associated with dose-dependent reductions in cirrhosis and hepatocellular carcinoma, among patients with hepatitis C virus: Results from ERCHIVES. Hepatology 2016;64:47–57. 10.1002/hep.28506 26891205 PMC4917438

[11] Mohanty A, Tate JP, Garcia-Tsao G. Statins are associated with a decreased risk of decompensation and death in veterans with hepatitis C-related compensated cirrhosis. Gastroenterology 2016;150:430–440. 10.1053/j.gastro.2015.10.007 26484707 PMC4727998

[12] Wang Y, Xiong J, Niu M, Chen X, Gao L, Wu Q, et al Statins and the risk of cirrhosis in hepatitis B or C patients: a systematic review and dose-response meta-analysis of observational studies. Oncotarget 2017;8:59666–59676. 10.18632/oncotarget.19611 28938670 PMC5601766

[13] Abraldes JG, Villanueva C, Aracil C, Turnes J, Hernandez-Guerra M, Genesca J, et al Addition of simvastatin to standard therapy for the prevention of variceal rebleeding does not reduce rebleeding but increases survival in patients with cirrhosis. Gastroenterology 2016;150:1160–1170. 10.1053/j.gastro.2016.01.004 26774179

[14] Lin SP, Long YM, Chen XH. The effects of statins on infections after stroke or transient ischemic attack: a meta-analysis. PLoS One 2015;10:e0130071 10.1371/journal.pone.0130071 26158560 PMC4497719

[15] Smit J, López-Cortés LE, Thomsen RW, Schonheyder HC, Nielsen H, Froslev T, et al Statin use and risk of community-acquired Staphylococcus aureus bacteremia: a population-based case-control study. Mayo Clin Proc 2017;92:1469–1478. 10.1016/j.mayocp.2017.07.008 28982483

[16] Magulick JP, Frei CR, Ali SK, Mortensen EM, Pugh MJ, Oramasionwu CU, et al The effect of statin therapy on the incidence of infections: a retrospective cohort analysis. Am J Med Sci 2014;347:211–6. 10.1097/MAJ.0b013e31828318e2 23426088 PMC3664245

[17] Hung TH, Lay CJ, Tseng CW, Tsai CC, Tsai CC. The effect of renal function impairment on the mortality of cirrhotic patients: a nationwide population-based 3-year follow-up study. PLoS One 2016;11:e0164824 10.1371/journal.pone.0164824 27631098 PMC5025109

[18] Hung TH, Tsai CC, Hsieh YH, Tsai CC, Tseng CW, Tsai JJ. Effect of renal impairment on mortality of patients with cirrhosis and spontaneous bacterial peritonitis. Clin Gastroenterol Hepatol 2012;10:677–681. 10.1016/j.cgh.2012.02.026 22391345

[19] Lewis JH, Mortensen ME, Zweig S, Fusco MJ, Medoff JR, Belder R, et al Efficacy and safety of high-dose pravastatin in hypercholesterolemic patients with well-compensated chronic liver disease: Results of a prospective, randomized, double-blind, placebo-controlled, multicenter trial. Hepatology 2007;46:1453–63. 10.1002/hep.21848 17668878

[20] Simon TG, King LY, Zheng H, Chung RT. Statin use is associated with a reduced risk of fibrosis progression in chronic hepatitis C. J Hepatol 2015;62:18–23. 10.1016/j.jhep.2014.08.013 25135867 PMC4272642

[21] Abraldes JG, Albillos A, Bañares R, Turnes J, Gonzalez R, Garcia-Pagan JC, et al Simvastatin lowers portal pressure in patients with cirrhosis and portal hypertension: a randomized controlled trial. Gastroenterology 2009;136:1651–1658. 10.1053/j.gastro.2009.01.043 19208350

[22] Motzkus-Feagans C, Pakyz AL, Ratliff SM, Bajaj JS, Lapane KL. Statin use and infections in veterans with cirrhosis. Aliment Pharmacol Ther 2013;38:611–618. 10.1111/apt.12430 23889738

[23] Kamal S, Khan MA, Seth A, Cholankeril G, Gupta D, Singh U, et al Beneficial effects of statins on the rates of hepatic fibrosis, hepatic decompensation, and mortality in chronic liver disease: a systematic review and meta-Analysis. Am J Gastroenterol 2017;112:1495–1505. 10.1038/ajg.2017.170 28585556

[24] Jepsen P, Ott P, Andersen PK, Sorensen HT, Vilstrup H. Clinical course of alcoholic liver cirrhosis: a Danish population-based cohort study. Hepatology 2010;51:1675–82. 10.1002/hep.23500 20186844

[25] D'Amico G, Garcia-Tsao G, Pagliaro L. Natural history and prognostic indicators of survival in cirrhosis: a systematic review of 118 studies. J Hepatol 2006;44:217–231. 10.1016/j.jhep.2005.10.013 16298014

